# Editorial: Insights in occupational health and safety: 2022

**DOI:** 10.3389/fpubh.2023.1257402

**Published:** 2023-09-05

**Authors:** Luigi De Maria, Antonio Caputi, Stefania Sponselli, Luigi Vimercati

**Affiliations:** Section of Occupational Medicine, Interdisciplinary Department of Medicine, University of Bari, Bari, Italy

**Keywords:** occupational health, occupational safety, prevention, worker, workplace

Occupational medicine is rapidly evolving from preventing health and safety risks in the workplace to promoting health for the total wellbeing of the worker according to the NIOSH “Total Worker Health” approach (1). This “*Insights in occupational health and safety: 2022*” Research Topic of 15 articles reflects this trend worldwide, with contributors from Asia, Europe, America, Africa, and Oceania. As for the 2021 edition (2), it includes forward-looking contributions focused on old and new occupational risk factors, recent advances and future perspectives in the field of Occupational Health and Safety.

From this perspective, Saberi et al. carried out an analysis to identify hot topics and Research Topics on occupational disease through the Web of Science from 1975 to 2021. The results showed occupational exposures, epidemiology, mental health, and respiratory diseases were the most important keywords used in these 45 years. In this regard, well-known forms of occupational and non-occupational exposure continue to pose a health risk in several countries of the world, including Italy (3, 4). Zhao et al. investigated the association of coal mine dust lung disease (CMDLD) with nodular thyroid disease in coal miners in China, finding that CMDLD was the strongest independent exposure risk factor for the development of nodular thyroid disease in coal miners. In the same country, Shi et al. conducted an observational trend study on global disease burden and trends of leukemia attributable to occupational risk from 1990 to 2019, finding a substantial reduction in leukemia due to occupational risks. On the other hand, Yuan et al. investigated occupational blood-borne pathogen exposure among dental nurses finding a high prevalence of sharp injuries in particular with syringe needle. Despite significant efforts over the past decade, job-related injuries are still one of the largest reasons contributing to disabilities and life-threatening conditions in developed and developing countries. Penney et al. showed that occupational fatality rates within the Australian commercial fishing industry are significantly higher than currently reported and recurring factors contributing to deaths at sea are unaddressed. Mekonnen et al. conducted a cross-sectional study among coffee processing industry workers in Ethiopia, highlighting a high prevalence of work-related disease symptoms and occupational injuries. Age group 30–39 and 40–49, income level, experience, smoking cigarette were significantly associated with the work-related symptom and training related to the job was significantly associated with occupational injuries. Another sector at high risk of occupational injuries in Ethiopia is construction sector. According to the study conducted by Yosef et al., the overall prevalence of occupational injuries among Bure industrial park construction workers was 39.4%. Being male, being married, no use of personal protective equipment, no training on occupational safety and not satisfied with the job were the factors associated with occupational injuries. Lee et al. compared the incidence of occupational diseases, avoidable hospitalization and all-cause death between firefighters and non-firefighters in Korea, from 2006 to 2005, finding that the standardized incidence ratios and hazard ratios for most diseases were high for firefighters. These studies show how occupational safety is still a highly relevant and serious issue worthy of academic attention and the research on strategies and policies to improve workers' safety behavior in reducing occupational injuries (Kim et al.) as well as workplace violence (Hu et al.) will become increasingly important in the future.

The “24-h society” that we have been approaching in recent years made night shift work a crucial factor in work organization, with well-established consequences on the workers' health and wellbeing. Regarding this issue Boini et al. evaluated the existing evidence on the effect of night-shift work on cardiovascular risk factors. After selection, 33 systematic reviews were included and the results confirmed an excess risk of diabetes, hypertension and overweight/obesity. Sleep duration is also independently associated with metabolic body size phenotypes (Wang et al.), while occupation type might be an independent factor in the development of diabetes (Habu et al.). Thus, occupational health physicians can give valuable help and support both to employers in planning the best possible shift schedule, and to workers in adopting the most appropriate personal coping strategies through ongoing health promotion interventions on modifiable lifestyle factors (5, 6).

May 11, 2023, marks the end of the COVID-19 public health emergency (7). In In the last 3 years significant increase in the prevalence of mental health disorders in different occupational settings has been associated with the COVID-19 pandemic, particularly for healthcare workers, who are at a high risk of exposure to infection and several psycho-social and work-related risk factors (8–15). Ito et al. explored mental health conditions among occupational therapists during the COVID-19 pandemic, demonstrating a direct link between therapists' mental health conditions and therapy quality. Edgelow et al. discussed the importance of adopting a broader conceptual approach to the study of public safety personnel mental health and proposed a novel model that highlights the need to consider the combined impacts of operational, organizational, and personal factors on public safety personnel mental health.

In conclusion, this “*Insights in occupational health and safety: 2022*” Research Topic includes a variety of occupational health and safety topics that show the new direction taken by research in this field, reflecting the contemporary holistic approach to worker wellbeing to help improve worker health and safety.

## Author contributions

LD: Conceptualization, Supervision, Writing—original draft, Writing—review and editing. AC: Conceptualization, Supervision, Writing—original draft, Writing—review and editing. SS: Conceptualization, Supervision, Writing—original draft, Writing—review and editing. LV: Conceptualization, Supervision, Writing—original draft, Writing—review and editing.
